# An Objective Method to Optimize the MR Sequence Set for Plaque Classification in Carotid Vessel Wall Images Using Automated Image Segmentation

**DOI:** 10.1371/journal.pone.0078492

**Published:** 2013-10-23

**Authors:** Ronald van ‘t Klooster, Andrew J. Patterson, Victoria E. Young, Jonathan H. Gillard, Johan H. C. Reiber, Rob J. van der Geest

**Affiliations:** 1 Division of Image Processing, Department of Radiology, Leiden University Medical Center, Leiden, The Netherlands; 2 University Department of Radiology, Cambridge University Hospitals NHS Foundation Trust, Cambridge, United Kingdom; College of Mechatronics and Automation, National University of Defense Technology, China

## Abstract

A typical MR imaging protocol to study the status of atherosclerosis in the carotid artery consists of the application of multiple MR sequences. Since scanner time is limited, a balance has to be reached between the duration of the applied MR protocol and the quantity and quality of the resulting images which are needed to assess the disease. In this study an objective method to optimize the MR sequence set for classification of soft plaque in vessel wall images of the carotid artery using automated image segmentation was developed. The automated method employs statistical pattern recognition techniques and was developed based on an extensive set of MR contrast weightings and corresponding manual segmentations of the vessel wall and soft plaque components, which were validated by histological sections. Evaluation of the results from nine contrast weightings showed the tradeoff between scan duration and automated image segmentation performance. For our dataset the best segmentation performance was achieved by selecting five contrast weightings. Similar performance was achieved with a set of three contrast weightings, which resulted in a reduction of scan time by more than 60%. The presented approach can help others to optimize MR imaging protocols by investigating the tradeoff between scan duration and automated image segmentation performance possibly leading to shorter scanning times and better image interpretation. This approach can potentially also be applied to other research fields focusing on different diseases and anatomical regions.

## Introduction

A multi-sequence MRI protocol is widely used to investigate the status of atherosclerosis in the carotid artery. Atherosclerosis is a progressive disease which, at an early stage, is characterized by vessel wall thickening causing outward remodeling, followed by narrowing of the lumen, and at a later stage by the formation of different plaque lesions inside the vessel wall [1]. Identification of vulnerable plaques, lesions with a high risk to rupture, before the development of cardiovascular events, is of high clinical relevance. These lesions are typically soft and contain a large lipid-rich necrotic core (LR/NC) or intraplaque hemorrhage (IPH) [2], which can both be identified by in-vivo MRI [3]. A typical MR imaging protocol for assessment of the carotid artery vessel wall consists of the application of multiple MR sequences to obtain information about the lumen morphology and a detailed characterization of the vessel wall [4].

An optimal in-vivo MR imaging protocol should incorporate contrast weightings that can separate the tissue of interest from surrounding structures, have a sufficiently high spatial resolution and have a short acquisition time. Since scanner time is limited, a balance has to be reached between the number of applied MR sequences and how well different plaque components can be distinguished from each other on the resulting images. Latest developments in this field include the design of new MR pulse sequences, adaption of existing MR sequences to higher field strength, design of specialized carotid coils and development of 3D isotropic imaging [5]. Consequently, objective evaluation of the resulting images is needed in order to determine the benefits of these continuous technological developments and the corresponding increase or decrease in scan time.

In an earlier work, Zhao et al. [6], presented a method to minimize the number of MR sequences and its impact on manual quantification of plaque lesions in vessel wall MR imaging studies. However, the proposed method has several drawbacks as the method is subjective because sets of different MR sequences are evaluated by the same readers introducing bias of the readers to their previous segmentation result. Also, the number of combinations of sequences that could be evaluated was limited. Cappendijk et al. [7] used a logistic regression model to determine the optimal MR weighting combination for plaque assessment enabling objective assessment of different combinations of sequences. However, assessment was limited to plaque identification; plaque volume was not taken into account. In addition, no relation was shown between the selected combination of MR sequences and the total scan duration. In another study by Liu et al. [8] an automated segmentation algorithm using pattern recognition techniques was applied. In that study the training of the classifier was performed on the same data as for which it was tested rendering subsequent results unreliable. Again, no relation was shown between the selected combination of MR sequences and the total scan duration. 

Accordingly, the purpose of this study was to develop an objective method to optimize the MR sequence set for classification of soft plaque in vessel wall MR images of the carotid artery with respect to segmentation accuracy and total scan duration. Instead of using a human observer to investigate different sets of MR sequences and their impact on plaque classification, automated image segmentation was employed which is inherently exhaustive and objective. The method was developed based on an extensive set of MR contrast weightings and corresponding manual segmentations of the vessel wall and soft plaque components, which were validated by histological sections. The proposed method is generic and can also be applied to other fields of research in which multi-spectral image data, such as multi-sequence MRI, is acquired and automated segmentation algorithms are available.

## Materials and Methods

### Ethics statement

The study was approved by The Cambridgeshire Three Research Ethics Committee. Written forms of informed consent were obtained from all subjects before imaging. Sharing of image data into the public domain was not permitted in the approved study protocol.

### Image data

Fifteen patients (11 male, age range 50-89 years), scheduled for carotid endarterectomy, were pre-operatively scanned using MRI. Color Doppler ultrasound was used for surgical screening to confirm the presence of stenosis >70%. Carotid specimens were retrieved after carotid endarterectomy. The endarterectomy specimens were fixed and cryosectioned to preserve the plaque components. Histological staining was performed with haematoxylin and eosin, elastic Van Gieson and nile red. The sections were digitized using a conventional microscope at 5X magnification (Leica DM LB2, Leica Microsystems, Wetzlar, Germany). The histological sections were visually matched with the MRI image data. For each patient at least one histological section was available which matched the imaging data. The matching sections served as ground truth for the manual image segmentation. 

MR imaging was performed on a 1.5T scanner (Signa HDx, GE Healthcare, Waukesha, WI) using a bilateral four-channel phased-array carotid coil (PACC, Machnet, Eelde, The Netherlands). The subject was positioned in the scanner with the carotid coils placed superficially over the anterior neck to cover the carotid artery centering on the carotid bifurcation. A vacuum constraining pillow system (Vac Lok Chushion, Oncology Systems Ltd, UK) was used to restrict head and neck movement.

In total six MR sequences were acquired from which nine contrast weightings were extracted. A 2D time-of-flight (TOF) angiography sequences was employed to identify the diseased segment and the position of the bifurcation. High-resolution multi-contrast axial images were obtained through the disease-affected section of the artery. The protocol included fat suppressed T_1_-weighted (T_1_W), dual echo T_2_/proton density-weighted (T_2_W/PDW) and short-tau inversion recovery (STIR) fast spin echo sequences using cardiac gated double inversion recovery preparation for blood suppression. Direct Thrombus Imaging (MRDTI) was performed using an inversion recovery T1 weighting prepared 3D fast spoiled gradient echo sequence [9]. The MRDTI sequence was acquired in the coronal plane to negate blood flow effects. A single-shot diffusion-weighted echo-planar imaging sequence was used to obtain T_2_W (b = 0 s/mm^2^) and diffusion-weighted (DW) images (b = 500 s/mm^2^) [10]. T_2_W and DW images were subsequently used to compute apparent diffusion coefficient (ADC) maps. The three resulting images are further labeled as DWT_2_, DWI, and ADC throughout the text. The image slices had a minimum coverage of 21 mm of the disease-affected section of the artery. A full list of the imaging parameters is shown in Table 1. The MR imaging protocol and subsequent post processing resulted in nine contrast weightings (TOF, T_1_W, T_2_W, PDW, STIR, MRDTI, DWT_2_, DWI, and ADC) which were used for further analysis.

**Table 1:** Carotid MR imaging pulse sequence parameters.

	**TOF**	**T_1_W**	**T_2_W/PDW**	**STIR**	**MRDTI**	**DWT2/DWI**
Acquisition	2D TOF - Axial	2D FSE - Axial	2D FSE - Axial	2D FSE - Axial	3D FSPGR - Coronal	2D EPI - Axial
FOV (cm)	22x22	10x10	10x10	10x10	10x10	16x16
pFOV	1	1	1	1	1	0.5
Matrix	512x512	256x256	256x256	256x256	160x160	128x128
NEX	1	2	2	2	1	16
TR (ms)	16.6	1R-R	2R-R	2R-R	5.7	2200
TE (ms)	4.1	7.7	99.7/7.7	99.7	2.6	~75
TI (ms)	-	-	-	150	19.0	-
Slice thickness (mm)	2	3	3	3	1	3
ETL	-	12	16	24	1	-
Fat suppression	No	Yes	Yes	Yes	No	Yes
In-plane resolution (mm)	0.86	0.39	0.39	0.39	0.625	1.25
Scan Time (min:sec) ^a^	0:48	5:22	8:24	5:36	2:09	4:42

a Scan times for either a 3D slab or multiple consecutive 2D slices compassing 21mm of carotid/plaque (assumes heart rate of 60bpm)

### Reference standard determined by manual image analysis

An experienced radiologist manually segmented the MR image data using the matching histological sections as ground truth to form the reference standard. The images from the nine contrast weightings were processed and manually segmented using the following steps:

1 T_1_W images were defined as reference images.
2 The other contrast weightings were processed to match the reference images resolution and geometry using multiplanar reconstruction.
3 The histological sections were visually matched with the image data. For each patient at least one matching histology section was available.
4 The lumen and outer contours were manually delineated on the T_1_W images by an experienced radiologist using CMRtools (Cardiovascular Imaging Solutions, London, UK). This software was also used, for subsequent steps 5 & 6, for the manual alignment of image slices and the delineation of plaque components.
5 The other MR sequences were manually aligned to the T_1_W image by translating the image slices in-plane to match the lumen and outer contours resulting in a set of aligned multi-contrast images.
6 The segmentation of the plaque components was performed by evaluating the images in the aligned set assisted by the available histology data. The plaque component contours were defined on the image where they could be seen most clearly. The expert manually delineated IPH, LR/NC and calcium regions.

Finally, the contours of IPH and LR/NC were combined and labeled as soft plaque. The manual image analysis resulted in an aligned set of vessel wall images including manual segmentations based on histology. The manual segmentation is further referred to as the *reference standard* in the remainder of the manuscript.

### Automated image segmentation using statistical pattern recognition

Several methods for segmentation of plaque components in the carotid artery have been described in literature, the majority employing statistical pattern recognition techniques [8,11–13]. Similarly, a pattern recognition system, developed using PRTools [14] and MATLAB (2009b, The MathWorks, Natick, MA, USA), was used to automatically classify the pixels within the vessel wall based on the different MR signal intensities. Vessel wall pixels were defined by the reference standard; the manually delineated contours of the lumen and outer wall. A standard PC was used to run the pattern recognition system software.

A pattern recognition system consists of a sensor that gathers the observations to be classified, a feature extraction mechanism that computes numeric or symbolic information from these observations, and a classifier that, based on these features, can classify the observations into different classes. In this study, the observations included the image slices of the nine contrast weightings and the manually segmented vessel wall contours. For each vessel wall pixel a number of features were extracted. These features contained normalized MR signal intensities and image gradient information. Based on these features, each observation was classified as soft plaque, defined as being LR/NC and/or IPH, or not being soft plaque. Calcium was not identified since the calcium regions were relatively small and the number of examples in the available image data was low.

#### Feature extraction

Prior to feature extraction, the vessel wall images were normalized by dividing the signal intensities by the median value of a 4 by 4 cm region of interest located at the lumen center [8]. This normalization step is required for comparing the different sequences of a single subject, as well as for inter-subject comparison. Subsequently, for each pixel inside the vessel wall the following features were extracted: the normalized signal intensity, zero-, first and second order derivatives at multiple scales (0.25, 0.5, 1.0 and 2.0 mm) from each of the vessel wall images. The zero-, first and second order derivative features were calculated using the RecursiveGaussianImageFilter of the open-source Insight Segmentation and Registration Toolkit (http://www.itk.org). The zero order derivatives are equivalent to blurred versions of the normalized signal intensities, the first and second order derivatives contain edge and texture information of the images.

#### Training and testing a classifier

The features of each observation served as input for a classifier. The classifier used is a Mahalanobis distance classifier, which is a type of Bayes classifier. A similar segmentation algorithm with a Bayes classifier was presented by Hofman et al. [12]. This is a supervised classifier that needs example observations and its corresponding classes in order to train the classifier. During the training phase, the features and the classes were used to learn statistics describing the example data. Once trained, the classifier can assign a class to unseen observations. Because of the relatively low number of patients, leave one out cross validation was used for training and testing the classifier. Data from one patient is used as the testing set, and the remaining patients are used as the training data. This is repeated such that each patient in the population is used once as the testing set.

### Evaluation of each combination of contrast weightings

**Figure 1:**
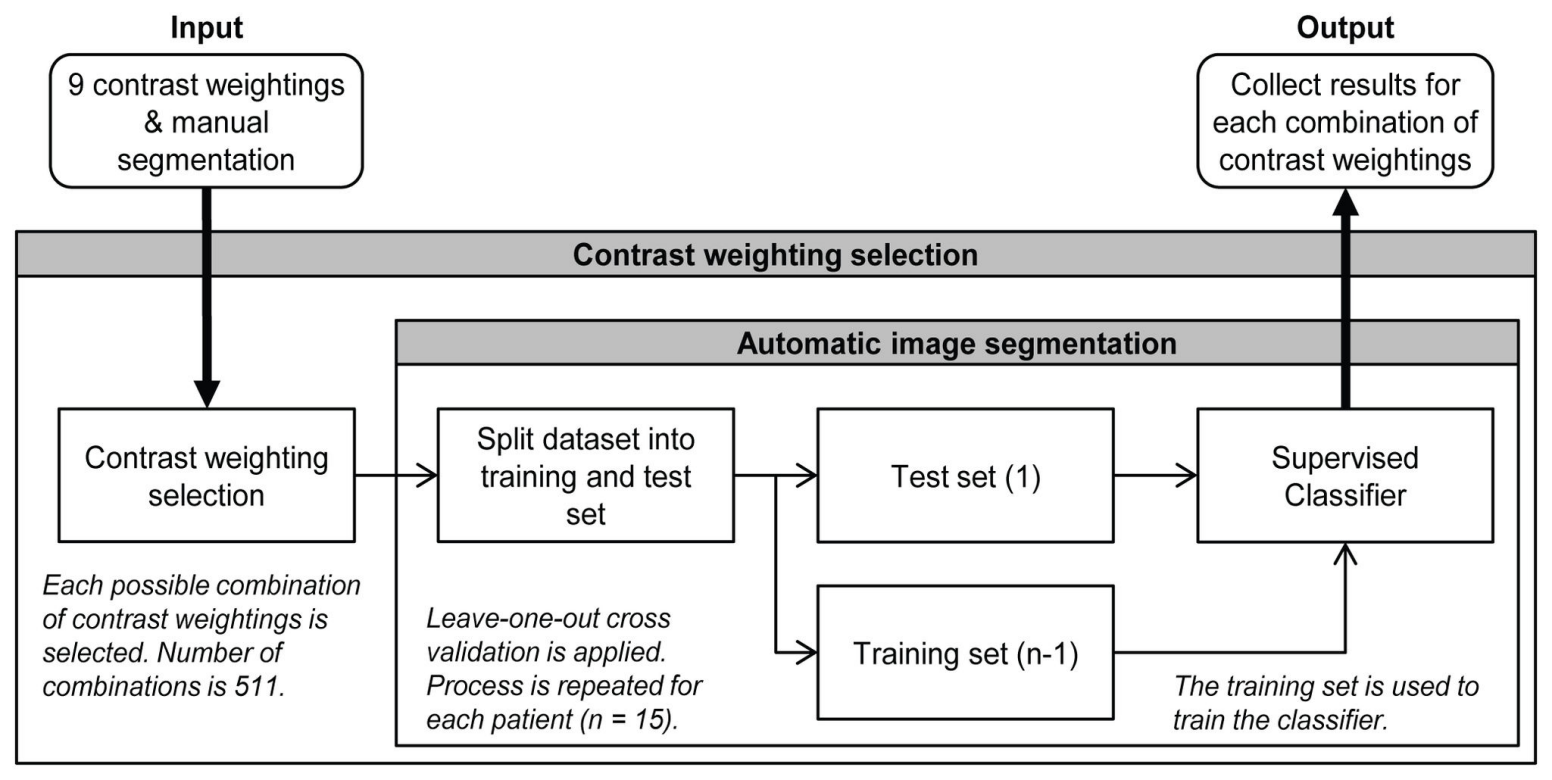
Scheme of the contrast weighting selection and the automatic image segmentation procedure. The procedure is started by the selection of a combination of contrast weightings. The automated image segmentation method is applied to that combination of contrast weightings and the reference standard which is based on the manual segmentation. The automated segmentation results are compared with the reference standard. This automated image segmentation procedure is then repeated for each contrast weighting selection and results of each combination are collected.

In this study nine contrast weightings from six different MR sequences were available. Each possible combination of contrast weightings was investigated by selecting a combination of weightings and applying the automated image segmentation method as shown in Figure 1. Besides the selection of the set of contrast weightings, no further feature selection was applied.

For each combination of contrast weightings the automatically segmented soft plaque volume per patient was compared to the reference standard using Pearson’s correlation. The correlation coefficient, p-value and confidence interval (CI) were calculated for each combination of contrast weightings. In addition, the time needed to acquire the MR sequences corresponding to the set of contrast weightings, was calculated.

## Results

Image data from 15 patients was analyzed by the manual observer and the automated image segmentation method. The presence and volumes of the plaque components in the patient population obtained by manual image analysis are given in Table 2.

**Table 2:** Plaque composition of patient population (n=15).

**Plaque component**	**Average volume ± SD (mm^3^)**
Calcium (n=5)	26.5 (± 15.5)
Hemorrhage (n=4)	75.6 (± 48.3)
Lipid (n=14)	142.0 (± 115.8)

SD, Standard Deviation.

**Figure 2:**
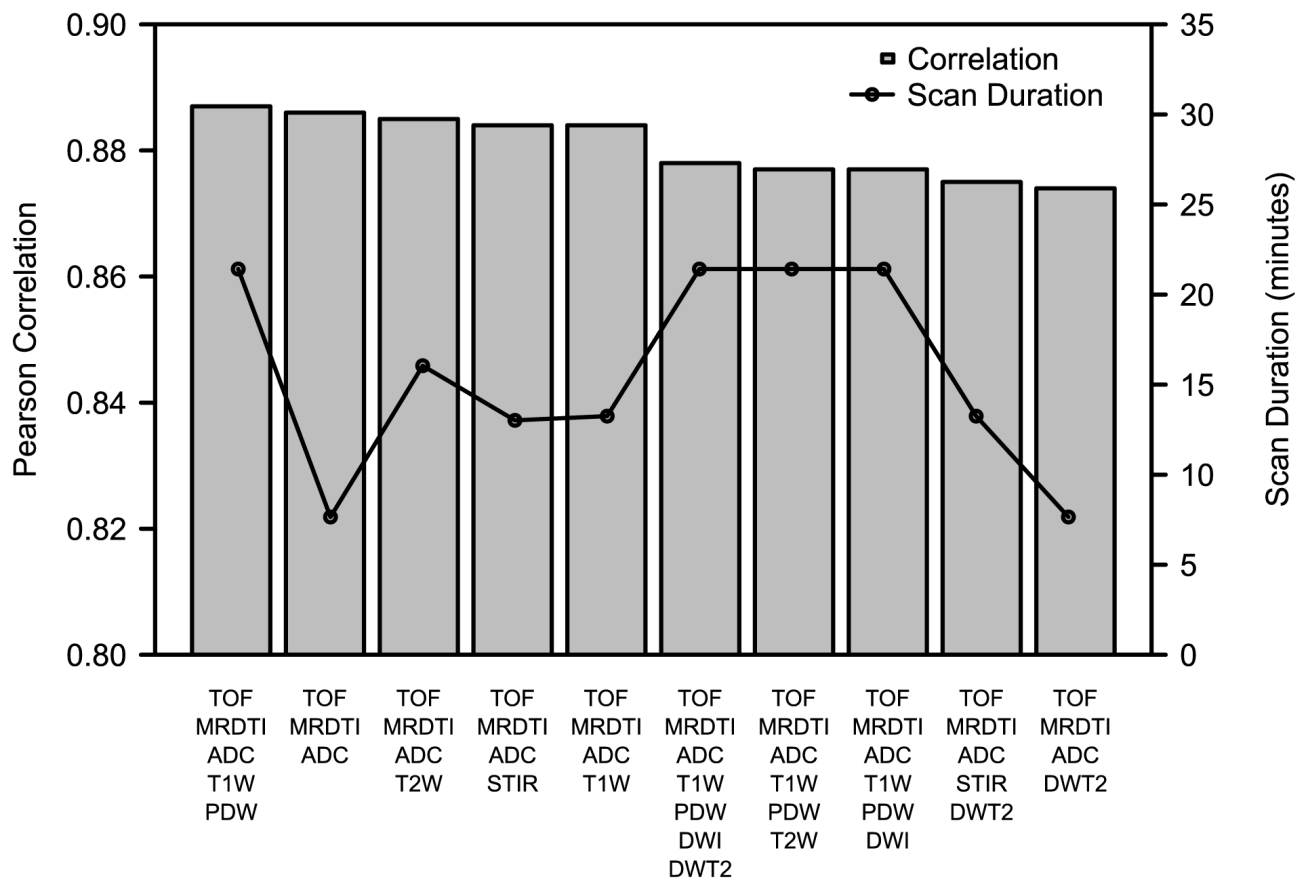
Comparison between automated segmentation performance, contrast weighting combinations and scan duration. The ten best contrast weighting combinations ranked by the correlation between the automated image segmentation and the reference standard are shown using a bar plot. Each bar represents one set of contrast weightings (contrast weightings are tiled horizontally on the horizontal axis). The total MR scan duration of each set is superimposed using a line plot.

In total 75 vessel wall slices (five slices per patient) were analyzed using the automated image segmentation method. The automated segmentation method was repeated for each combination of contrast weightings. The number of combinations which were tested was 511 (2^9^ - 1). The selected contrast weightings, correlations, CIs, p-values and corresponding scan durations are available in the Table S1. Figure 2 shows the correlation and total MR scan duration for the ten best contrast weighting combinations. 

**Figure 3:**
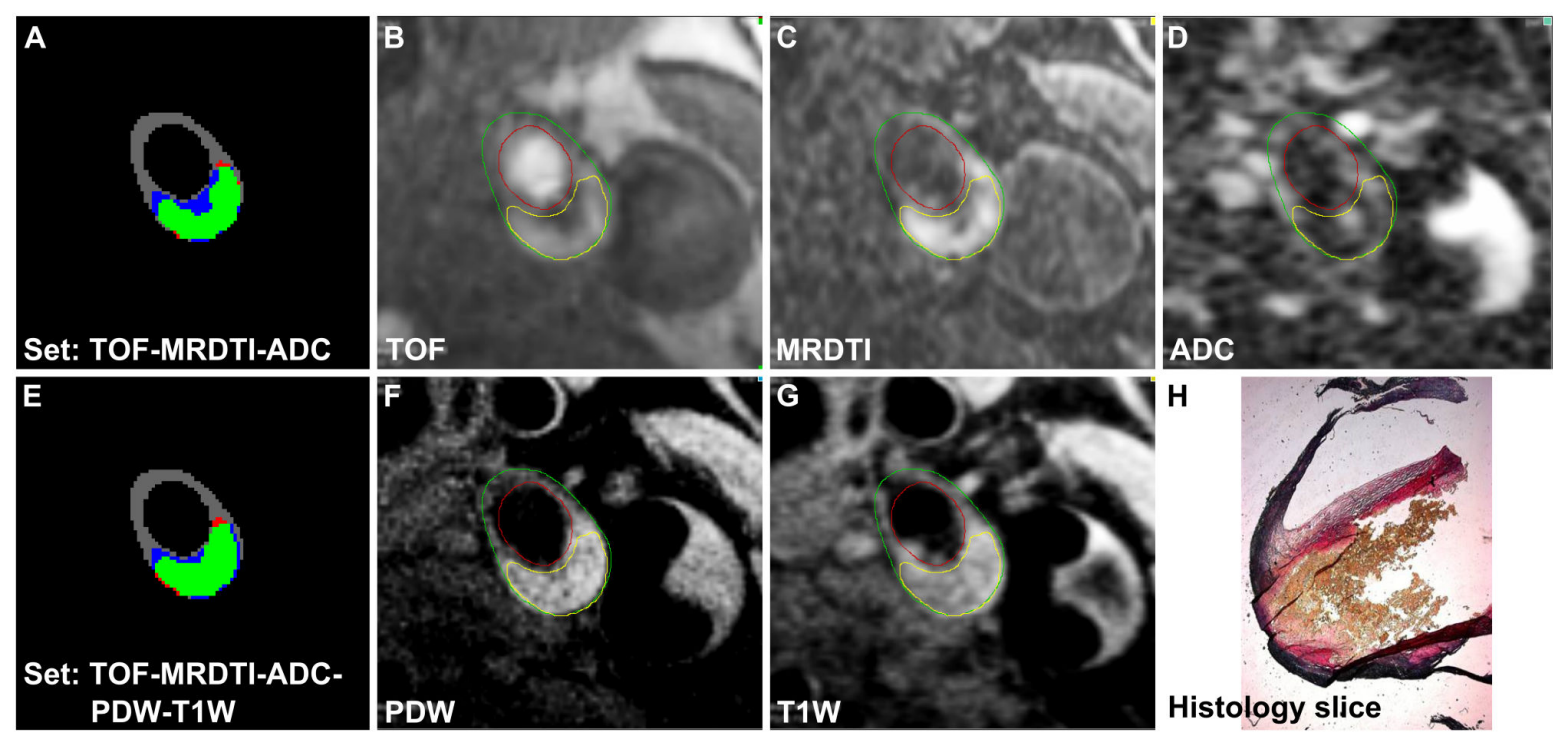
Automated segmentation results for two sets of contrast weightings including MR images and histology. A) segmentation result for the contrast weighting set TOF-MRDTI-ADC showing the overlap between the automated segmentation method and the reference standard (green: true positive lesion, blue: false negative lesion, red: false positive lesion), B) TOF image, C) MRDTI image, D) ADC image, E) segmentation result for the set TOF-MRDTI-ADC-PDW-T1W, F) T1W image, G) PDW image, and H) the matching histological slice with elastic Van Gieson staining. The contours of the reference standard are overlaid on the different MR images (panels B,C,D,F,G). The lumen and vessel wall are depicted by the red and green contour, soft plaque is depicted by the yellow contour. The yellow colour in the histological slice in panel H is consistent with the presence of lipid.

In Table 3 the results are ordered based on the number of contrast weightings used. The highest correlation is achieved by selecting five weightings. However, while using the combination of TOF, MRDTI and ADC weightings, the correlation is almost similar to the highest correlation and the scan time is reduced by more than 60%. Figure 3 shows the difference in plaque segmentation result between using 3 and 5 weightings for one image slice of the dataset.

**Table 3:** Correlation between the automated image segmentation and the reference standard ranked by number of contrast weightings.

**Contrast weightings**	**Number of weightings**	**Pearson Correlation [CI]**	***p*-value**	**MR scan duration [min]**
TOF	1	0.711 [0.312-0.897]	*p* = 0.003	0.8
TOF-MRDTI	2	0.860 [0.622-0.953]	*p* < 0.001	3.0
TOF-MRDTI-ADC	3	0.886 [0.683-0.962]	*p* < 0.001	7.7
TOF-MRDTI-ADC-T2W	4	0.885 [0.682-0.961]	*p* < 0.001	16.1
TOF-MRDTI-ADC-PDW-T1W	5	0.887 [0.688-0.962]	*p* < 0.001	21.4
TOF-MRDTI-ADC-PDW-T1W-T2W	6	0.877 [0.663-0.959]	*p* < 0.001	21.4
TOF-MRDTI-ADC-PDW-T1W-DWI- DWT2	7	0.878 [0.665-959]	*p* < 0.001	21.4
TOF-MRDTI-ADC-PDW-T1W-DWI-DWT2-T2W	8	0.855 [0.611-0.951]	*p* < 0.001	21.4

CI, Confidence Interval.min, minutes.

## Discussion

An objective method to optimize the MR imaging protocol for plaque classification in vessel wall images using an automated classifier was presented. Evaluation of the results from nine contrast weightings showed the tradeoff between scan duration and automated segmentation performance. The presented method has potential for application in a clinical setting, or may be of value when developing new imaging protocols for clinical research.

The automated image segmentation method is based on pattern recognition and uses only the vessel wall images and histology based manual segmentations in order to achieve a direct interpretation of the informational content of these images and the segmentation performance. While morphology was used to delineate the boundaries of the vessel wall, no morphological features were used in the automated image segmentation algorithm to ensure that the segmentation results are directly related to the image information of the contrast weightings. Also, no feature selection or post processing was used. The performance of the algorithm was in line with previous studies [8,12]. Furthermore, our approach is free of the constraints imposed by previous studies which sought to define a minimal number of sequences using manual segmentation [6,8], specifically the number of combinations that could be evaluated, observer bias and the lack of an independent training set.

Performance of the automated segmentation method increased by the number of selected contrast weightings with an optimum at five weightings. Selection of more than five weightings showed a decrease in performance. Such a behavior is typical for a pattern recognition system; adding information to the system by selecting more features increases the performance, but at a certain point the performance stabilizes or decreases because the extra features do not contain relevant or contradicting information. The first selected contrast weighting was the TOF image. Both LR/NC and IPH can be identified from this weighting [2] explaining why this weighting was the first one to be selected by the presented method. Next, the MRDTI weighting was added to the set; the MRDTI weighting was reported to be sensitive for the detection of IPH [9]. The third selected weighting was the ADC image, which can be used to distinguish LR/NC [10] from other plaque components and fibrous tissue. Addition of the other contrast weightings provided only a small improvement which, in our opinion, was not relevant.

This study is subject to a number of limitations. No distinction was made between IPH and LR/NC. However, if more image data is available, the method can be extended by either applying the method to each plaque component independently or by pooling the segmentation results for the different plaque components. In the first case, the optimal sequence set for a single plaque component can be investigated. In the second case, the set of sequences is evaluated with respect to all plaque components. The reference standard was acquired using manual segmentation by a radiologist and is therefore not completely objective. The manual segmentation process was assisted by histology reducing the effect of human variability in the reference standard. There were no matching histology sections for all image slices. In cases where no matching histology section was available, the nearest histology section was interpreted in order to perform the delineation of the vessel wall and plaque components combining the standard assumptions of the relative contrast weighting of plaque components [4]. Due to the limited size of the dataset, leave-one-out cross validation was used for the training and testing of the automated segmentation algorithm. This method provides an approximately unbiased estimate of the classification performance. A larger dataset, which can be split into a training set and an independent testing set, can provide a more reliable estimate of the classification performance. The presented soft plaque correlation measurements have wide confidence intervals. Because of these wide intervals it is not possible to indicate whether or not a particular set of vessel wall images is significantly better than another set of images. However, the different correlations can still be compared and the scan duration should be considered in the interpretation of the results.

In conclusion, a method was presented to objectively evaluate the MR sequence protocol for the detection of the soft plaque in MR vessel wall images of the carotid artery. The presented approach allows development and optimization of MR imaging protocols by investigating the tradeoff between scan duration and automated segmentation performance possibly leading to shorter scanning times and better image interpretation. This approach can potentially also be applied to other research fields focusing on different diseases and anatomical regions.

## Supporting Information

Table S1 **Contrast weightings combinations and corresponding correlations, CIs, p-values and scan durations.**
(XLS)
